# A Monte Carlo Method for Calculating Lynden-Bell Equilibrium in Self-Gravitating Systems

**DOI:** 10.3390/e25101379

**Published:** 2023-09-25

**Authors:** Tarcísio N. Teles, Calvin A. F. Farias, Renato Pakter, Yan Levin

**Affiliations:** 1 Grupo de Física de Feixes, Universidade Federal de Ciências da Saúde de Porto Alegre (UFCSPA), Porto Alegre 90050-170, RS, Brazil; tarcisio.nteles@gmail.com; 2 Instituto de Física, Universidade Federal do Rio Grande do Sul (UFRGS), Caixa Postal 15051, Porto Alegre 91501-970, RS, Brazil; calvin.farias@ufrgs.br (C.A.F.F.); pakter@if.ufrgs.br (R.P.)

**Keywords:** long range, Lynden-bell, Monte Carlo, core halo, 05.20.-y, 05.70.Ln, 05.70.Fh

## Abstract

We present a Monte Carlo approach that allows us to easily implement Lynden-Bell (LB) entropy maximization for an arbitrary initial particle distribution. The direct maximization of LB entropy for an arbitrary initial distribution requires an infinite number of Lagrange multipliers to account for the Casimir invariants. This has restricted studies of Lynden-Bell’s violent relaxation theory to only a very small class of initial conditions of a very simple waterbag form, for which the entropy maximization can be performed numerically. In the present approach, an arbitrary initial distribution is discretized into density levels which are then evolved using an efficient Monte Carlo algorithm towards the final equilibrium state. A comparison is also made between the LB equilibrium and explicit Molecular Dynamics simulations. We find that for most initial distributions, relaxation is incomplete and the system is not able to reach the state of maximum LB entropy. In particular, we see that the tail of the stationary particle distribution is very different from the one predicted by the theory of violent relaxation, with a hard cutoff instead of an algebraic decay predicted by LB’s theory.

## 1. Introduction

Understanding the mass distribution in self-gravitating systems has remained a longstanding challenge that has intrigued researchers for over seven decades [1,2,3]. The resolution of this enigma holds the potential to illuminate many theoretical puzzles, such as the physical mechanisms underlying the regularities observed in the light profile of elliptical galaxies and the mass distribution in dark matter halos.

Classical simulations conducted by Navarro, Frenk, and White [4,5] produced density profiles of dark matter halos that current theories struggle to explain. Numerous references [6,7,8,9,10,11,12,13] explore the scope of this problem, extending from the foundations of statistical mechanics to the large-scale evolution of the universe [14].

In contrast to systems with short-range interactions, which equilibrate through collisional processes, long-range systems relax primarily through collective effects arising from the interactions of individual particles with the entire system. The long-range interacting systems exhibit intriguing features such as: ensemble inequivalence [15], temperature inversion effects [16], and the emergence of long-lived quasi-stationary states [17]. These unique characteristics set them apart from their short-range counterparts and make them fascinating subjects for study and exploration.

In the thermodynamic limit, interparticle correlations are absent in systems with long-range interactions, and the *N*-particle distribution function, $f^{N}\left({\vec{q}}_{1},\ldots ,{\vec{q}}_{N},{\vec{p}}_{1},\ldots ,{\vec{p}}_{N},t\right)$, can be factorized into a product of one-particle distribution functions, $f(\vec{q},\vec{p},t)$,

(1) $$f^{N}\left({\vec{q}}_{1}\;,\ldots \;,{\vec{q}}_{N}\;,{\vec{p}}_{1}\;,\ldots \;,{\vec{p}}_{N}\;,t\right)=\prod_{k=1}^{N}f\left({\vec{q}}_{k},{\vec{p}}_{k},t\right)$$

where $\left({\vec{q}}_{k},{\vec{p}}_{k}\right)$ are the generalized coordinate and momentum of a particle in the phase space. This factorization reduces the 2dN-dimensional phase space to the phase space of a single particle. The evolution of the one-particle distribution function is governed by the Vlasov equation [18]:

(2) $$\frac{\partial f}{\partial t}+\vec{p}\cdot \frac{\partial f}{\partial \vec{q}}-\frac{\partial \psi (\vec{q},t)}{\partial \vec{q}}\cdot \frac{\partial f}{\partial \vec{p}}=0\;,$$

where $\psi \left(\vec{q},t\right)=\;\int \int \;\phi (|\vec{q}-\vec{q}{\;}^{\prime }\left|\right)f\left(\vec{q}{\;}^{\prime },\vec{p}{\;}^{\prime },t\right)d\vec{q}{\;}^{\prime }d\vec{p}{\;}^{\prime }$ denotes the mean-field potential associated with the pair potential $\phi (|\vec{q}-\vec{q}{\;}^{\prime }|)$.

The evolution of $f(\vec{q},\vec{p},t)$ in the phase space is similar to that of an incompressible fluid—maintaining a constant local density along the flow. Furthermore, the Vlasov dynamics have an infinite number of invariants known as the Casimirs [19,20,21,22]—any local functional of the distribution function is a Casimir invariant. In particular, a hyper volume of any density level η of the initial distribution function is a Casimir invariant of the Vlasov dynamics,

(3) $$C\left(\eta ,t\right)=\int \delta \left[f\left(\vec{q}\;,\vec{p}\;,t\right)-\eta \right]d^{d}\vec{q}\;d^{d}\vec{p}=C\left(\eta ,0\right).$$

The total mass *M* and the energy of the system $E_{0}$ are also conserved quantities:

(4) $$\int d^{d}\vec{q}\;d^{d}\vec{p}\;f\left(\vec{q},\vec{p},t\right)=M\;,$$

(5) $$\int d^{d}\vec{q}\;d^{d}\vec{p}\;\left(\frac{\vec{p}{\;}^{2}}{2m}+\frac{\psi (\vec{q})}{2}\right)\;f\left(\vec{q}\;,\vec{p}\;,t\right)=E_{0}\;.$$

In the case of non-neutral plasmas, *M* would refer to the total charge of the system [23,24]. Finally, both linear and angular momentum are also conserved quantities. However, for symmetric initial distributions studied in the present paper, their initial values can be set to zero.

Unlike the collisional Boltzmann equation, which has the Maxwell distribution as the global attractor, the Vlasov equation does not have an attractor. The relaxation to the stationary state of the Vlasov equation proceeds through the process of filamentation. In fact, on a fine-grained scale the relaxation never stops—only on the coarse-grained scale can we say that the system has reached a stationary state. Any distribution of the form $f_{ss}\left(\vec{q}\;,\vec{p}\right)\equiv f\left[\epsilon_{ss}\left(\vec{q}\;,\vec{p}\right)\right]$, where $\epsilon_{ss}\left(\vec{q}\;,\vec{p}\right)=\vec{p}{\;}^{2}/\left(2m\right)+\psi_{ss}\left(\vec{q}\right)$ is the single-particle energy, is a potential candidate to describe the stationary state (ss). However, predicting *a priori* the specific distribution to which a system will relax starting from an arbitrary initial condition remains a challenge [1].

Long before the empirical work of Navarro, Frenk, and White [4,5], Lynden-Bell (LB) introduced a very elegant statistical theory of relaxation in systems with long-range interactions, with particular emphasis on self-gravitating systems [7]. This theory became known as the Theory of Violent Relaxation. By discretizing the initial particle distribution into multiple levels within the context of Vlasov dynamics, LB assumed that the evolution of the density levels is ergodic and that there is a complete mixing of the one-particle distribution function in the phase space. Under these assumptions, LB argued that on a coarse-grained scale, the system will evolve to the maximum entropy state allowed by the conservation of mass, energy, and the Casimir invariants. In particular, for an initial distribution of one level—the waterbag distribution—the coarse-grained stationary distribution function is found to be,

(6) $${\bar{f}}_{ss}\left(\vec{q},\vec{p}\right)=\frac{\eta_{0}}{e^{\beta [\epsilon_{ss}\left(\vec{q}\;,\vec{p}\right)-\gamma ]}+1},$$

where γ and β are the Lagrange multipliers determined by the conserved quantities (Equations (4) and (5)), and the potential is calculated self-consistently by solving the Poisson equation,

(7) $${\vec{\nabla }}_{d}{\;}^{2}\psi_{ss}\left(\vec{q}\right)=C_{d}\int {\bar{f}}_{ss}\left(\vec{q}\;,\vec{p}\right)d^{d}\vec{p}\;,$$

where $C_{d}$ is a constant associated with the dimensionality of the system and ${\vec{\nabla }}_{d}$ represents the Laplacian operator in the *d*-dimensional space [25].

The form of the distribution proposed by Lynden-Bell (LB) (6) exhibits similarities with the Fermi–Dirac distribution. However, it is essential to emphasize that we are dealing with classical “particles”. The exclusion principle associated with fermions, in this case, has a parallel with the constraint that the evolution of the distribution function, according to Vlasov dynamics, is analogous to that of an incompressible fluid, meaning that different levels of the distribution function cannot overlap at any point in the phase space.

**Figure 1:**
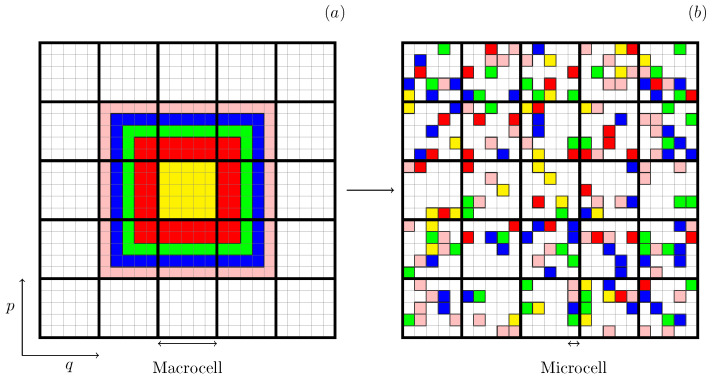
Schematic representation of a two-dimensional phase space containing μ=5×5 macrocells, with each macrocell containing ν=25 microcells. The one-particle distribution function, initially with l=5 levels, is represented by different colors and evolves over time from (**a**) to (**b**). In this representation, each microcell can either remain empty or be filled with a given level $\eta_{i}$, while the coarse-grained distribution function is determined at the level of each macrocell.

At the core of LB’s theory is the fundamental assumption that violent relaxation induces a rapid phase-space mixing of the levels of the initial distribution function. On a coarse-grained scale, the one-particle distribution function is expressed as an average over the density levels present inside the macrocell, as depicted in Figure 1.

We stress that the equilibrium distribution function proposed by LB exists only at the level of macrocells, as there is no increase of entropy at the microcell level—fine-grained Gibbs entropy is one of the Casimir invariants of the Vlasov dynamics. This is similar to what occurs in short-range systems when analyzed in terms of the *N*-particle distribution function in a 2dN-dimensional phase space. The increase of Gibbs entropy takes place only at the coarse-grained macrocell level since at the microcell level, the Gibbs entropy remains constant due to Liouville’s theorem. In both cases, this problem can be circumvented by using Boltzmann entropy, which is defined at the coarse-grained level [26,27]. Nevertheless, it is worth emphasizing that LB’s statistical approach is equivalent to the traditional method of maximizing the Gibbs entropy, provided that all the Casimirs are taken into account [22].

When attempting to apply Lynden-Bell’s statistical theory to real-world systems that involve multiple levels *l* of the initial one-particle distribution functions, a significant challenge arises due to the highly nonlinear nature of the problem. In this case, *l* equations, similar to Equation (4), are required, one for each level, which substantially increases the computational complexity. Consequently, obtaining solutions under these general initial conditions becomes impractical for large *l*, making it challenging to achieve accurate and reliable results. Moreover, the system’s sensitivity to initial parameters further compounds the difficulties, hindering the discovery of the final equilibrium state. As a result, the application of Lynden-Bell’s theory has been primarily confined to single-level distribution functions [28]. While this restricted set of initial conditions offers some valuable insights into certain aspects of self-gravitating systems, its generality is limited—preventing us from making conclusions about what will happen with much more complex continuous initial distributions. To address these challenges, the primary objective of the present paper is to propose a Monte Carlo (MC) approach that efficiently leads to the equilibrium state corresponding to the maximum of LB entropy, with all the relevant constraints taken into account.

## 2. Monte Carlo Algorithm

We start by discretizing the initially continuous distribution $f_{0}\left(\vec{q},\vec{p}\right)$ into *l* levels [29], where each level *i* has a density $\eta_{i}$, with 1≤i≤l. We designate level l+1 as the empty cell, with $\eta_{l+1}=0$. Thus, the initial distribution can be expressed as:

(8) $$f_{0}^{j}=\sum_{i=1}^{l+1}n_{i}^{j}\frac{\eta_{i}}{\nu }$$

Here, $n_{i}^{j}$ represents the number of microcells containing level *i* inside the macrocell *j*, centered on $\left(\vec{q},\vec{p}\right)$. The total number of macrocells is defined as $\mu =G_{q}\times G_{p}$, where $G_{q}$ is the number of position grids, and $G_{p}$ is the number of momentum grids. At t=0, each macrocell centered on $\left(\vec{q},\vec{p}\right)$ has only one level $\eta_{i}$, with the value closest to that of $f_{0}\left(\vec{q},\vec{p}\right)$. Since each microcell can contain only one density level, we have a bound $0\leq n_{i}^{j}\leq \nu$, where ν is the number of microcells in a macrocell, see Figure 1.

**Figure 2:**
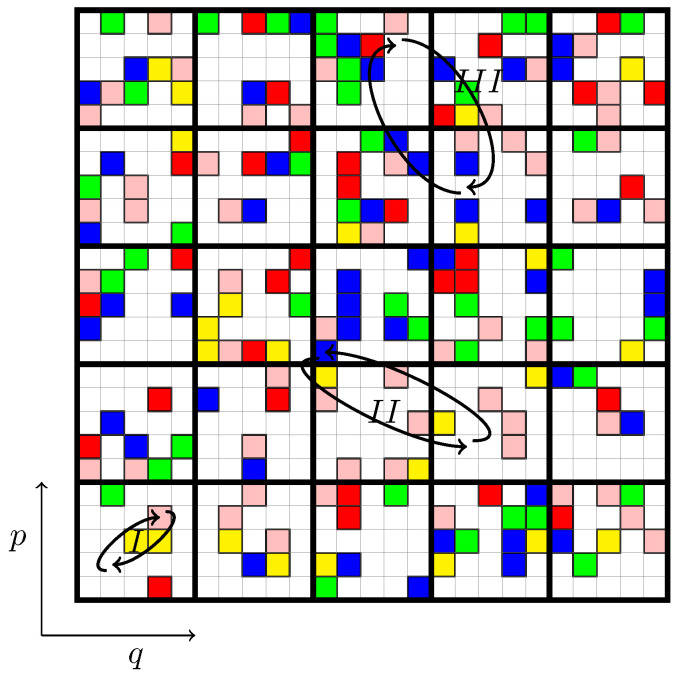
Schematic representation of the exchange process in the occupation of microcells in phase space. Processes II and III illustrate the exchange of two microcells inside different macrocells, while process *I* represents the exchange within the same macrocell. Process III, on the other hand, depicts an exchange between different microcells but with the same density, where the empty cell is considered to have null density, and due to this, it is an irrelevant process.

The key aspect of the Monte Carlo (MC) technique is to evolve the system to an equilibrium state consistent with the macroscopic constraints, such as the total energy, mass, momentum, and the conservation of the total volume occupied by each density level. In equilibrium, the system can explore all microstates that are consistent with the constraints. To force the system towards equilibrium, MC moves must respect the detailed balance condition:

(9) P(o)π(o→n)=P(n)π(n→o),

where (*o*) and (*n*) refer to old and new configurations corresponding to two different microstates. P is the equilibrium probability that the system is in a given microstate. As can be seen in Figure 1 and Figure 2, all microstates on the energy shell are equally probable, so that P=1 for any microstate.

The transition probability π consists of two steps:

(10) π(o→n)=α(o→n)×acc(o→n),

where α(o→n) represents the proposal of a trial move from one microstate (*o*) to another (*n*), and acc(o→n) denotes the acceptance probability associated with deciding whether to accept or reject this trial move.

In the MC simulation, various trial moves are possible, one simple example being the exchange of levels between two microcells. In this case, it is important to note that α(o→n)=α(n→o) since we can always swap levels between any two arbitrary microcells (on the energy shell) (see Figure 2). As a result, the acceptance probabilities for both transitions are equal:

(11) $$\frac{acc(o\to n)}{acc(n\to o)}=1,$$

meaning that a swap between one or more pairs of microcells is always allowed, provided it conserves the total energy and other macroscopic constraints.

Such a brute force approach is very inefficient since only microstates that are confined to the energy shell can be moved. Most moves will be rejected because they do not conserve the total energy of the system. To enhance the efficiency of such microcanonical MC methods, Creutz [30] proposed to relax the constraint of exact energy conservation—allowing the total energy of the system to slightly fluctuate close to its initial value. This is achieved by introducing an additional degree of freedom for a system to store energy. Creutz called this additional degree of freedom a “demon”. The first move is only allowed if it lowers the energy of the system. The excess energy is then stored inside the demon. In the following move, the difference δE is examined. If the trial move lowers the energy, it is accepted and the energy gained from such a move is stored inside the demon. On the other hand, if δE>0, the move is accepted *only if* the demon has sufficient energy in store to compensate for the system’s energy gain. If it does, the move is accepted, and the quantity δE is subtracted from the demon’s energy store. The MC dynamics is illustrated in Figure 2.

For application to LB statistics, a simple MC algorithm described above is still very inefficient. The position of each microcell has to be stored in an array and track must be kept of all the swaps between the different microcells. Clearly, since the energy and the coarse-grained distribution function are calculated on the level of macrocells, the details of which microcell inside the macrocell is occupied by the specific density levels are irrelevant. The only relevant information is how many density levels of each type are present inside the macrocell. Based on this observation, we now introduce a more efficient approach for performing MC at the level of macrocells.

As before, the phase space is divided into μ macrocells, each containing a fixed number ν of microcells that can either be occupied by a certain level *i* of the initial distribution or left empty, as illustrated in Figure 1. Trial moves now involve adding or removing levels within a specific macrocell, as demonstrated by the processes II and III of Figure 2. Only process II is relevant, since, in process III, the proposed exchange is between equal levels and does not affect the coarse-grained distribution function.

To illustrate the algorithm, let us consider an exchange of two distinct levels, denoted i=a and i=b, between two different macrocells, labeled j=A and j=B, as shown in processes II of Figure 2. In this trial move, we will remove a level *a* from the macrocell *A* and place it into macrocell *B*, and similarly, we will remove a level *b* from the macrocell *B* and place it into macrocell *A*. This is summarized as follows:

In macrocell *A*:

(12) $$\begin{array}{ccc} n_{a}^{A} & \to n_{a}^{A}-1,\;n_{b}^{A} & \to n_{b}^{A}+1. \end{array}$$

In macrocell *B*:

(13) $$\begin{array}{ccc} n_{b}^{B} & \to n_{b}^{B}-1,\;n_{a}^{B} & \to n_{a}^{B}+1. \end{array}$$

Note that the redistribution of levels within the same macrocell does not affect the energy of the system, since it is calculated only at the level of macrocells. The total degeneracy *W* for a given distribution of levels $\left\{n_{i}^{j}\right\}$ across all the macrocells is, therefore,

(14) $$W=\prod_{j=1}^{\mu }\frac{\nu !}{\prod_{i=1}^{l+1}n_{i}^{j}!}.$$

We should observe that differently from LB’s original work, we adopt Gibbs counting, and treat the same density levels as indistinguishable [7]. In practice, this does not affect any of the final results, and makes the combinatorics simpler.

The probability of finding the system in its old configuration, characterized by the macrocell *A* with $\left(n_{a}^{A}\;,n_{b}^{A}\right)$, and the macrocell *B* with $\left(n_{a}^{B}\;,n_{b}^{B}\right)$, will be proportional to:

(15) $$P\left(o\right)\propto \cdots \frac{\nu !}{n_{a}^{A}!n_{b}^{A}!}\frac{\nu !}{n_{a}^{B}!n_{b}^{B}!}\cdots$$

while after performing the swap, the probability of finding the system in its new configuration, characterized by the macrocell *A* with $\left(n_{a}^{A}-1\;,n_{b}^{A}+1\right)$, and the macrocell *B* with $\left(n_{a}^{B}+1\;,n_{b}^{B}-1\right)$, will be proportional to:

(16) $$P\left(n\right)\propto \cdots \frac{\nu !}{\left(n_{a}^{A}-1\right)!\left(n_{b}^{A}+1\right)!}\frac{\nu !}{\left(n_{a}^{B}+1\right)!\left(n_{b}^{B}-1\right)!}\cdots .$$

Note that the choice of density levels that are being proposed for a swap is completely unbiased, performed with equal probability among all the levels that are present within the two macrocells. Therefore, the probability of choosing a trial move α(o→n)=α(n→o)=1. Substituting this into the detailed balance equation, Equation (9), we find that the acceptance probabilities in the forward and reverse directions must satisfy:

(17) $$\frac{acc(o\to n)}{acc(n\to o)}=\frac{P(n)}{P(o)}=\frac{n_{a}^{A}\;n_{b}^{B}}{\left(n_{b}^{A}+1\right)\left(n_{a}^{B}+1\right)}.$$

Since the maximum acceptance probability is one, following Metropolis [31], we conclude that:

(18) $$acc\left(o\to n\right)=\min\left(1,\frac{n_{a}^{A}\;n_{b}^{B}}{\left(n_{b}^{A}+1\right)\left(n_{a}^{B}+1\right)}\right).$$

In this new approach, the trial moves within the same macrocell, depicted by process *I* of Figure 2, are no longer necessary since their mixing and degeneracy are taken into account exactly by the combinatorial factors in Equations (15) and (16). This represents a significant gain compared to the original MC method. To summarize: we randomly select two out of the μ macrocells in the system, along with two levels from the available levels within these macrocells. Next, we generate a random number ζ, uniformly distributed between 0 and 1, and then check if ζ<acc(o→n). If this condition is met, we accept the swap; otherwise, we reject it. Note, the choice of trial moves remains symmetric, α(o→n)=α(n→o), as we continue to choose the levels for a swap independent of their occupation of the macrocell. Although this MC algorithm represents a significant improvement over the brute force MC, the fact that ζ<1 implies that many of the proposed moves will be rejected. This unnecessary inefficiency arises when we encounter cases where $n_{a}^{A}∼n_{b}^{B}∼1$. In such situations, there are probably other levels (different from *a* and *b*) that are more appropriate for a swap. This suggests that the choice of proposed moves should not be completely random, but biased [32] toward levels that have larger occupations within the macrocells *A* and *B*.

To implement this bias, we now select the levels based on their occupation in the macrocell, rather than choosing them uniformly as we did before. The probability of choosing level *a* inside the macrocell *A* is taken to be proportional to its occupation within the macrocell $n_{a}^{A}/\nu$, and similarly for level *b* inside the macrocell *B*. The biased choice then:

(19) $$\alpha \left(o\to n\right)=\frac{n_{a}^{A}n_{b}^{B}}{\nu^{2}},$$

Note that now, α(o→n)≠α(n→o) since the choice in the reversed direction is:

(20) $$\alpha \left(n\to o\right)=\frac{\left(n_{a}^{A}+1\right)\left(n_{b}^{B}+1\right)}{\nu^{2}}.$$

To make this discussion clearer, consider the central macrocell of Figure 2, and let us call it *A*. Now, let us associate colors to represent different levels: yellow for level 1, red for level 2, green for level 3, blue for level 4, pink for level 5, and empty spaces for level 6. Therefore, in macrocell *A*, we have the following distribution: $n_{1}^{A}=0$, $n_{2}^{A}=0$, $n_{3}^{A}=2$, $n_{4}^{A}=6$, $n_{5}^{A}=1$, and $n_{6}^{A}=16$, totaling ν=25.

To randomly select a level within macrocell *A*, we generate a random number between 0 and ν=25. If the generated number falls between 0 and 1, we choose level 5 (pink). If it falls between 1 and 3, the chosen level will be 3 (green). If it falls between 3 and 9, the chosen level will be 4 (blue). Finally, if the number falls between 9 and 25, the chosen level will be 6, representing an empty space. This is similar for macrocell *B*. The probability of selecting a level *a* in macrocell *A* and a level *b* in macrocell *B* is then given by Equation (19). Substituting Equations (19) and (20) together with Equations (15) and (16) into the expression for the detailed balance Equation (9), we obtain:

(21) $$\frac{acc(o\to n)}{acc(n\to o)}=\frac{\alpha (n\to o)}{\alpha (o\to n)}\frac{P(n)}{P(o)}=1.$$

Therefore, as long as the levels proposed for a swap are chosen according to their occupation inside the two macrocells, the swap move is always accepted—assuming, of course, that the move either lowers the energy of the system or that the demon has enough energy in store to compensate it [30]. This makes the MC algorithm extremely efficient. Below, we summarize the MC algorithm:

1. Start the system in a given initial configuration and set the demon energy $E_{d}$ to 0.
2. Calculate the total energy.
3. Choose two distinct macrocells (the first one can be chosen following some order, and the second one randomly) labeled *A* and *B*.
4. Select two distinct levels proportionately to their presence inside the two macrocells (biased selection). This is done by generating two random numbers between 0 and ν, and determining which intervals defined by $\left\{n_{i}^{A}\right\}$ and $\left\{n_{i}^{B}\right\}$ they fall into.
5. Perform the trial moves and calculate the change in the total energy of the system:
  $$\delta E=\left(\eta_{a}-\eta_{b}\right)\times \left(\epsilon_{B}-\epsilon_{A}\right).$$
6. If $\delta E\leq E_{d}$, accept the move and update the energy of the reservoir as $E_{d}=E_{d}-\delta E$. Otherwise, reject the move and go back to step 3.
7. Go through all μ macrocells once (this defines one MC step).
8. Recalculate the potential $\psi (\vec{q})$ by numerically integrating Equation (7) and return to step 2.

We start by testing our MC algorithm on simple one- and two-level waterbag distributions for which the LB entropy function can be maximized exactly. We undertake this investigation within the framework of the one-dimensional self-gravitating model (ODSGM), a model that has been extensively studied in the field of stellar dynamics since the seminal works of Lecar [33] and Hohl [34,35], up to the more recent works of Miller [36,37,38]. It has also found applications in cosmological models explored by Joyce and collaborators [39,40,41]. Despite its simplification compared to real three-dimensional gravity, the ODSGM already contains the main aspects of the gravitational problem—long-range potential [28,42], collective motion, particle-wave interactions, which lead to collisionless relaxation [23,43,44], etc. For this study, we opt to use the notation (x,v) instead of $\left(\vec{q},\vec{p}\right)$ to specify the position of a particle in the phase space. A notable practical advantage of the ODSGM is the absence of singular particle–particle interaction since the two-body potential is $\phi \left(x,x^{\prime }\right)=\left|x-x^{\prime }\right|$, facilitating both theory and simulations [39,40].

**Figure 3:**
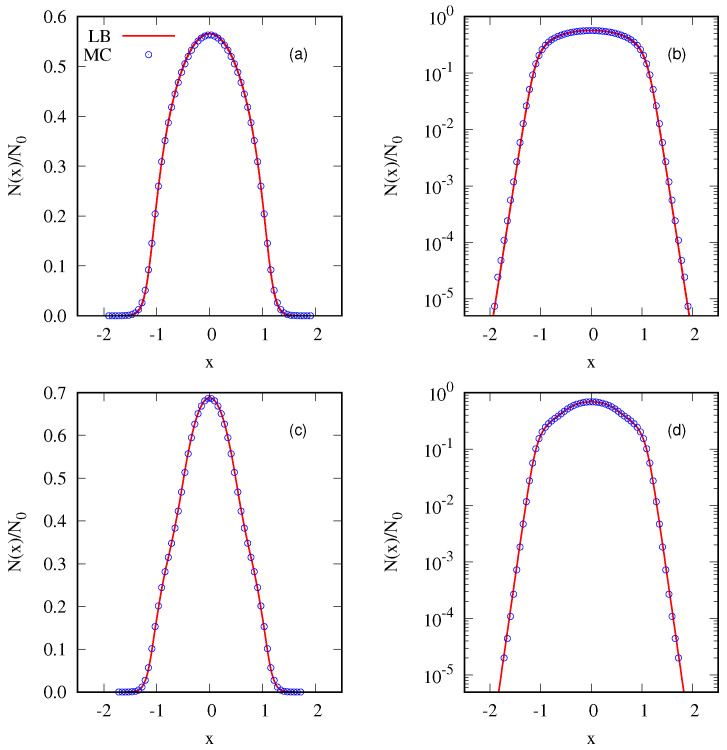
Equilibrium state for the initial one–level (waterbag) distribution, as defined in Equation (22). In (**a**), the density in space is shown on a linear scale, whereas (**b**) employs a semilog scale. (**c**,**d**) show the equilibrium state for a two–level distribution described by Equation (23). (**c**) is presented using a linear scale, while (**d**) utilizes the semilog scale for the density in space. Solid lines are calculated using exact LB entropy maximization, while symbols are the results of MC simulation. In MC, we used μ=64×64 and ν=128 for both distributions.

**Figure 4:**
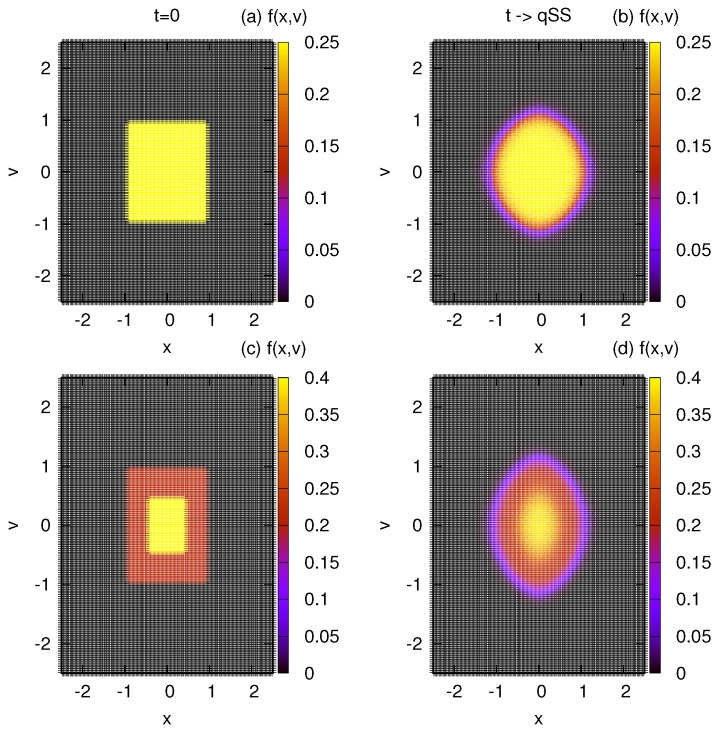
Evolution of the distribution function in MC. (**a**,**b**) show the initial and final distributions for the one–level distribution of Equation (22). (**c**,**d**) show the initial and final states, respectively, for the two–level distribution of Equation (23). The parameters are set as ν=128 and μ=64×64.

We begin by studying the equilibrium state to which ODSGM relaxes from an initial single-level waterbag distribution:

(22) $$f_{wb_{1}}\left(x,v\right)=\eta_{1}\Theta (x_{1}-|x|)\Theta (v_{1}-|v|),$$

where Θ(x) represents the Heaviside function, $x_{1}=v_{1}=1$ and $\eta_{1}=1/4$. The symmetry of the distribution results in a null total linear *momentum*, with the only conserved quantity beside the total mass M=1 being the total energy $E_{0}$. The LB equilibrium is obtained by numerically solving Equations (6) and (7) with constraints given by Equations (4) and (5). A perfect agreement between the exact (numerical) solution and the MC simulation is demonstrated in Figure 3, validating the algorithm presented above. We next perform a similar calculation for a two-level waterbag distribution:

(23) $$\begin{array}{c} \begin{array}{cc} f_{wb_{2}}\left(x,v\right) & =\left(\eta_{1}-\eta_{2}\right)\Theta (x_{1}-|x|)\Theta (v_{1}-\left|v|\right)\\ & \;+\eta_{2}\Theta (x_{2}-|x|)\Theta (v_{2}-|v|). \end{array} \end{array}$$

where $x_{1}=v_{1}=1/2$, $x_{2}=v_{2}=1$, $\eta_{1}=0.4$ and $\eta_{2}=0.2$. Once again, we obtain perfect agreement with simulation results; Figure 3 and Figure 4.

**Figure 5:**
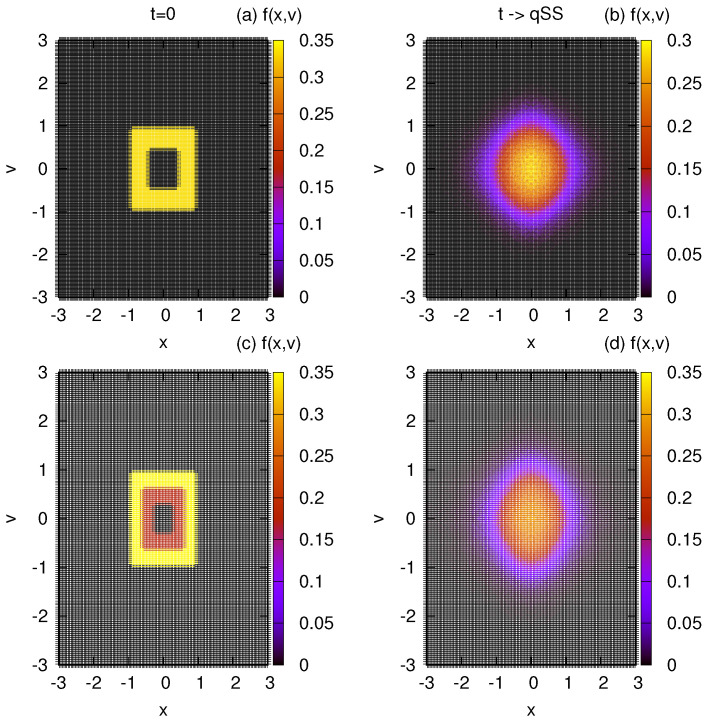
Evolution of the distribution function in MC. (**a**,**b**) show the initial and final distributions for the one–level initial condition, Equation (24). (**c**,**d**) show the initial and final distributions, respectively, for the two-level initial condition of Equation (25). The MC parameters are: ν=128 and μ=64×64.

**Figure 6:**
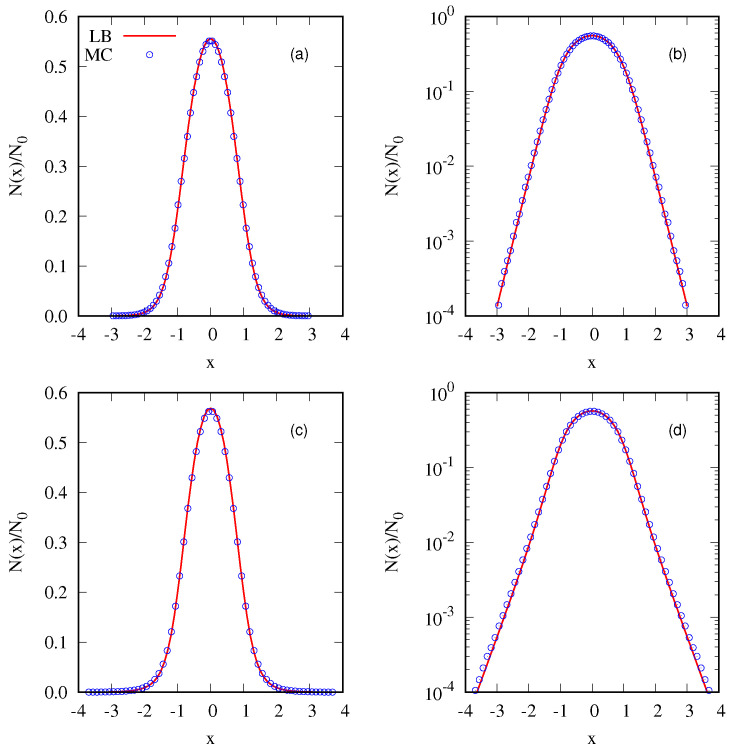
Equilibrium state for the initial one–level (waterbag) distribution, as defined in in Equation (24). In (**a**), the particle density distribution is shown on a linear scale, whereas (**b**) shows the same on a semilog scale. For the two–level distribution described by Equation (25), (**c**) shows the final equilibrium state on a linear scale (**c**), and (**d**) shows the same on a semilog scale. Solid lines are calculated using exact LB entropy maximization, while symbols are the results of MC simulation. The MC used μ=64×64 and ν=128 for both distributions.

We next look at the one-level and two-level distributions, with zero density at the center, see Figure 5. For the one-level distribution with the hole at the center we use:

(24) $$\begin{array}{cc} f_{wb_{3}}\left(x,v\right) & =\eta_{1}\Theta (x_{1}-|x|)\Theta (v_{1}-\left|v|\right)\\ \; & -\eta_{1}\Theta (x_{2}-|x|)\Theta (v_{2}-|v|). \end{array}$$

where $x_{1}=v_{1}=1$, $x_{2}=v_{2}=1/2$ and $\eta_{1}=1/3$. For a two-level waterbag:

(25) $$\begin{array}{cc} f_{wb_{4}}\left(x,v\right) & =\eta_{2}\Theta (x_{3}-|x|)\Theta (v_{3}-\left|v|\right)\\ \; & -\left(\eta_{2}-\eta_{1}\right)\Theta (x_{2}-|x|)\Theta (v_{2}-\left|v|\right)\\ \; & -\eta_{1}\Theta (x_{1}-|x|)\Theta (v_{1}-\left|v|\right) \end{array}$$

where $x_{1}=v_{1}=1/3$, $x_{2}=v_{2}=2/3$, $x_{3}=v_{3}=1$, $\eta_{1}=1/6$, and $\eta_{2}=1/3$. We observe that for these initial distributions, the LB equilibrium states do not have holes in the center—instead, we see that the central region is most densely populated according to LB theory (Figure 6). Again, we observe a perfect agreement between the numerical LB entropy maximization and our MC simulations.

**Figure 7:**
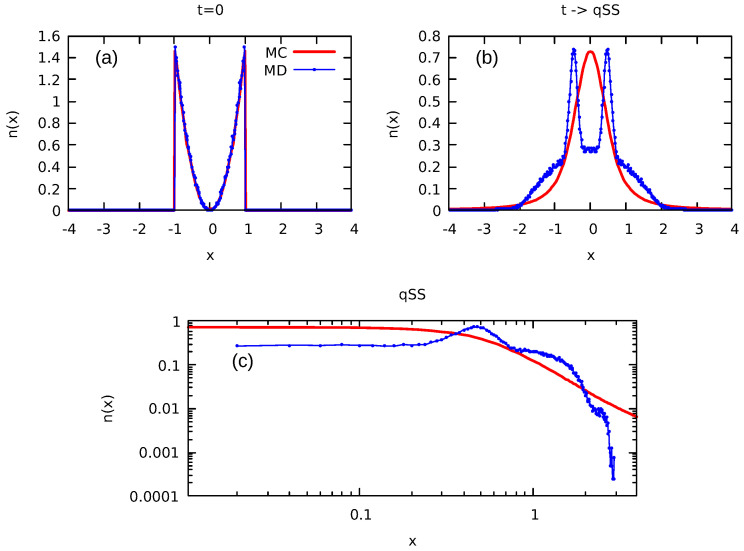
Initial parabolic distribution described by Equation (26) discretized in l=20 levels. In the MC simulations we use ν=1024, μ=256×256. (**a**) shows the initial particle density distribution and (**b**) shows the final equilibrium distribution. (**c**) shows the equilibrium distribution on a logarithmic scale. MD simulations were performed with $N=2\times 10^{5}$ particles.

**Figure 8:**
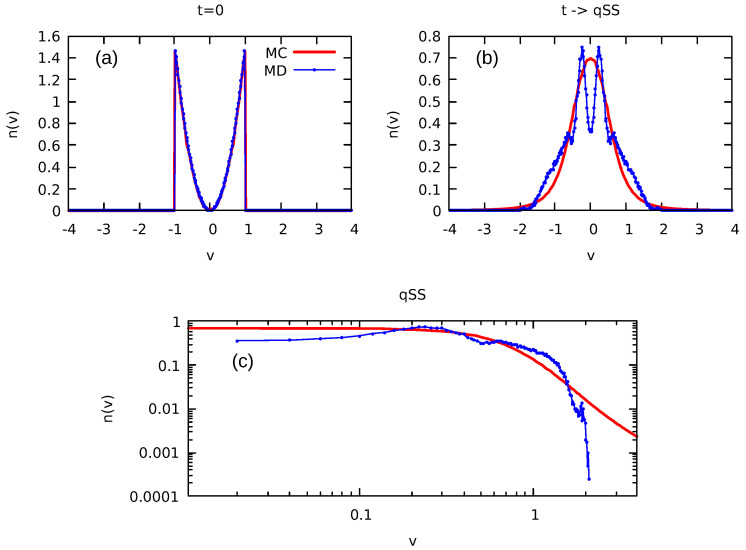
Initial parabolic distribution described by Equation (26). All parameters are the same as in Figure 7. (**a**) illustrates the initial velocity distribution and (**b**) shows the final velocity distribution in LB equilibrium. (**c**) shows the velocity distribution on a logarithmic scale. MD simulations were performed with $N=2\times 10^{5}$ particles.

Considering that the validity of our algorithm is now fully established, we are now poised to leverage this approach for the analysis of continuous initial distributions, for which the numerical solution of the LB equations is no longer possible. As a demonstration of the applicability of the MC method, we consider an initial distribution that has a parabolic profile in both position and velocity:

(26) $$f_{cont}\left(x,v\right)=\frac{9x^{2}v^{2}}{4x_{m}^{3}v_{m}^{3}}\;\Theta (x_{m}-|x|)\;\Theta (v_{m}-|v|),$$

with particles confined in a rectangle of $|x_{m}|\leq 1$ and $|v_{m}|\leq 1$. To perform MC simulations, this distribution is discretized into l=20 levels, randomly generated from the range of 0 to $f_{max}$, where $f_{max}$ corresponds to the highest value of the initial distribution Equation (26). The results of the equilibrium particle distribution obtained using our MC algorithm are presented in Figure 7 and Figure 8.

**Figure 9:**
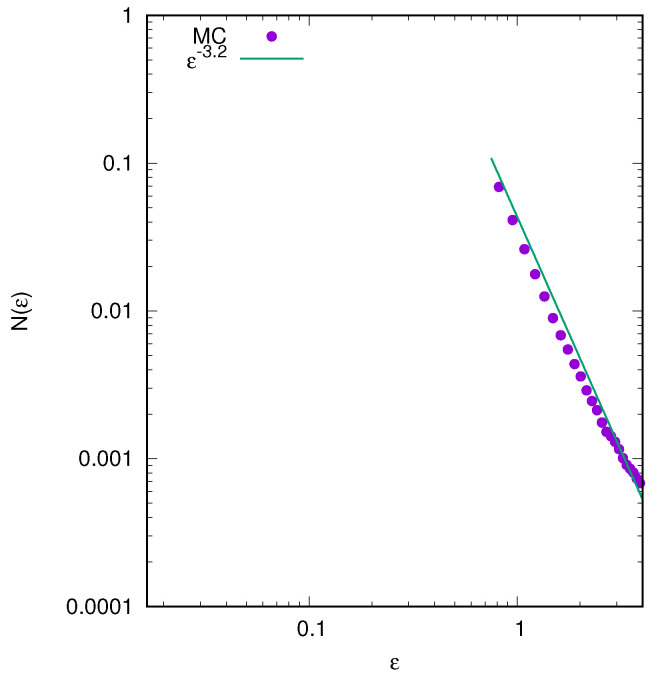
Particle energy distribution N(ϵ) at equilibrium obtained using MC simulations. After initial exponential decay, we observed a power–law decay at high energies. All parameters are the same as in Figure 7.

It is interesting to compare the equilibrium distributions predicted by the LB theory with the results of molecular dynamics (MD) simulations. In the MD simulations, we start by distributing *N* particles according to Equation (26) and then evolve the system using Newton’s equations of motion until a stationary state is reached. Figure 7 and Figure 8 present both the initial and final stationary particle distributions calculated using LB theory (MC simulations) and MD. We observe that while the initial distributions are identical, the final stationary state to which the system evolves is very different from the predictions of LB theory. While LB theory produces a slowly decaying tail in the particle density distribution function—see Figure 7, Figure 8 and Figure 9—MD has a very sharp decay consistent with the resonant core-halo theory [45]. Furthermore, the MD distribution in the core region clearly shows incomplete relaxation—the particle density remains depleted in the core, while LB theory predicts a complete population inversion in which higher density levels predominantly occupy the core region. These findings perhaps are not very surprising in view of the earlier work on gravitational systems and on systems with long-range interactions in general [46,47]. Still, there was an expectation that the increased complexity of the multilevel distribution might help the system to relax to the LB equilibrium. Instead, we see that relaxation remains incomplete.

## 3. Conclusions

In this paper, we presented a Monte Carlo approach that allows us to obtain the LB equilibrium state for an arbitrary initial particle distribution. For such continuous distributions, direct maximization of LB entropy requires an infinite number of Lagrange multipliers and is not practical for systems of interacting particles. This restricted the applicability of Lynden-Bell’s theory to initial conditions of a very simple waterbag form, for which the entropy maximization could be performed numerically. In the present approach, an arbitrary initial distribution is discretized into density levels which are then evolved using an efficient Monte Carlo algorithm towards the state that corresponds to the LB equilibrium.

It is interesting to note that for continuous initial distributions, the LB equilibrium particle distribution found using MC simulations shows the presence of algebraically decaying tails [48,49]. We examined a range of diverse initial conditions for the distributions described by Equation (26), as illustrated in Figure 9. In all cases, the energy distribution shows an initially exponential decay, followed by a power-law tail with exponents appearing in the range of [2.7–3.5] for the distributions analyzed in the present work.

Unfortunately, we find that the equilibrium particle distribution predicted by the LB theory is very different from what is observed in the actual MD simulations. While LB theory produces a slowly decaying tail in the particle density distribution function, MD simulations show a very sharp decay of the particle distribution—see Figure 7 and Figure 8—consistent with the resonant core-halo theory [45]. In the core-halo theory, the mechanism for particle evaporation arises from the resonant interactions between individual particles and the collective oscillations. The resonant (separatrix) orbit controls the maximum energy that any particle can gain from the collective oscillations [1]. Furthermore, particle evaporation results in Landau damping, which eventually kills all oscillations, producing a stationary mean-field potential. When this happens, the system becomes integrable [46,47,50], all the evolution ceases, and it remains trapped in a stationary state. Comparison of the MC results with the explicit MD simulations indicates that for continuous initial distributions, the same scenario plays out. Unfortunately, increasing the complexity of initial distributions does not help systems relax to LB equilibrium.

## Data Availability

Not applicable.

## References

[1] Levin Y., Pakter R., Rizzato F.B., Teles T.N., Benetti F.P.C. (2014). Nonequilibrium statistical mechanics of systems with long-range interactions. Phys. Rep..

[2] Barnes E.I., Williams L.L.R. (2011). Entropy Production in Collisionless Systems. I. Large Phase-space Occupation Numbers. Astrophys. J..

[3] Hjorth J., Williams L.L.R. (2010). Statistical Mechanics of Collisionless Orbits. I. Origin of Central Cusps in Dark-Matter Halos. Astrophys. J..

[4] Navarro J.F., Frenk C.S., White S.D.M. (1995). Simulations of X-ray clusters. Mon. Not. R. Astron. Soc..

[5] Navarro J.F., Frenk C.S., White S.D.M. (1996). The Structure of Cold Dark Matter Halos. Astrophys. J..

[6] Camm G.L. (1950). Self-gravitating Star Systems. Mon. Not. R. Astron. Soc..

[7] Lynden-Bell D. (1967). Statistical mechanics of violent relaxation in stellar systems. Mon. Not. R. Astron. Soc..

[8] Cuperman S., Goldstein S., Lecar M. (1969). Numerical experimental check of Lynden-Bell statistics-II. The core-halo structure and the role of the violent relaxation. Mon. Not. R. Astron. Soc..

[9] Hénon M. (1971). The Monte Carlo method. Astrophys. Space Sci..

[10] Shu F.H. (1978). On the statistical mechanics of violent relaxation. Astrophys. J..

[11] White S.D.M., Narayan R. (1987). Maximum entropy states and the structure of galaxies. Mon. Not. R. Astron. Soc..

[12] Mathur S.D. (1990). Existence of oscillation modes in collisionless gravitating systems. Mon. Not. R. Astron. Soc..

[13] Saslaw W.C. (2000). The Distribution of the Galaxies: Gravitational Clustering in Cosmology.

[14] Padmanabhan T. (1990). Statistical mechanics of gravitating systems. Phys. Rep..

[15] Teles T.N., Fanelli D., Ruffo S. (2014). Ensemble inequivalence in systems with wave-particle interaction. Phys. Rev. E.

[16] Teles T.N., Gupta S., Di Cintio P., Casetti L. (2016). Temperature inversion in long-range interacting systems. Phys. Rev. E.

[17] Chakhmakhchyan L., Teles T.N., Ruffo S. (2017). Ensemble inequivalence and absence of quasi-stationary states in long-range random networks. J. Stat. Mech..

[18] Braun W., Hepp K. (1977). The Vlasov dynamics and its fluctuations in the 1/N limit of interacting classical particles. Commun. Math. Phys..

[19] Chavanis P.-H. (2006). Coarse-grained distributions and superstatistics. Phys. A.

[20] Chavanis P.-H. (2022). Kinetic theory of collisionless relaxation for systems with long-range interactions. Phys. A.

[21] Ewart R.J., Brown A., Adkins T., Schekochihin A.A. (2022). Collisionless relaxation of a Lynden-Bell plasma. J. Plasma Phys..

[22] Rocha Filho T.M., Figueiredo A., Amato M.A. (2005). Entropy of Classical Systems with Long-Range Interactions. Phys. Rev. Lett..

[23] Levin Y., Pakter R., Teles T.N. (2008). Collisionless Relaxation in Non-Neutral Plasmas. Phys. Rev. Lett..

[24] Teles T.N., Pakter R., Levin Y. (2009). Relaxation and emittance growth of a thermal charged-particle beam. Appl. Phys. Lett..

[25] Levin Y. (2002). Electrostatic correlations: From plasma to biology. Rep. Prog. Phys..

[26] Lebowitz J. (1973). Ergodic theory and statistical mechanics of non-equilibrium processes. Nonlinear Probl. Phys. Sci. Biol..

[27] Lebowitz J. (1999). Microscopic origins of irreversible macroscopic behavior. Physica.

[28] Campa A., Dauxois T., Ruffo S. (2009). Statistical mechanics and dynamics of solvable models with long-range interactions. Phys. Rep..

[29] Fracassi Farias C.A., Pakter R., Levin Y. (2023). Euler fluid in two dimensions: Statistical approach. Phys. Rev. E.

[30] Creutz M. (1983). Microcanonical Monte Carlo Simulation. Phys. Rev. Lett..

[31] Metropolis N., Rosenbluth A.W., Rosenbluth M.N., Teller A.H., Teller E. (1953). Equation of State Calculations by Fast Computing Machines. J. Chem. Phys..

[32] Frenkel D., Smit B. (2001). Understanding Molecular Simulation.

[33] Lecar M. (1966). A one-dimentional self-gravitating stellar gas. The Theory of Orbits in the Solar System and in Stellar Systems.

[34] Hohl F., Feix M.R. (1967). Numerical Experiments with a One-dimensional Model for a self-gravitating Star System. Astrophys. J..

[35] Hohl F., Campbell J.W. (1968). Statistical Mechanics of a Collisionless Self-Gravitating System. Astron. J..

[36] Miller B.N., Rouet J.-L. (2010). Cosmology in one dimension: Fractal geometry, power spectra and correlation. J. Stat. Mech..

[37] Reidl C., Miller B. (1995). Population dependence of early relaxation. Phys. Rev. E.

[38] Miller B.N., Manfredi G., Pirjol D., Rouet J.-L. (2023). From chaos to cosmology: Insights gained from 1D gravity. Class. Quantum Gravity.

[39] Joyce M., Worrakitpoonpon T. (2011). Quasistationary states in the self-gravitating sheet model. Phys. Rev. E.

[40] Joyce M., Worrakitpoonpon T. (2010). Relaxation to thermal equilibrium in the self-gravitating sheet model. J. Stat. Mech..

[41] Joyce M., Sicard F. (2011). Non-linear gravitational clustering of cold matter in an expanding universe: Indications from 1D toy models. Mon. Not. R. Astron. Soc..

[42] Roule M., Fouvry J.-B., Pichon C., Chavanis P.-H. (2022). Long-term relaxation of one-dimensional self-gravitating systems. Phys. Rev. E.

[43] Levin Y., Pakter R., Rizzato F.B. (2008). Collisionless relaxation in gravitational systems: From violent relaxation to gravothermal collapse. Phys. Rev. E..

[44] Teles T.N., Levin Y., Pakter R., Rizzato F.B. (2010). Statistical mechanics of unbound two-dimensional self-gravitating systems. J. Stat. Mech..

[45] Teles T.N., Levin Y., Pakter R. (2011). Statistical mechanics of 1D self-gravitating systems: The core—Halo distribution. Mon. Not. R. Astron. Soc..

[46] Ribeiro-Teixeira A.C., Benetti F.P.C., Pakter R., Levin Y. (2014). Ergodicity breaking and quasistationary states in systems with long-range interactions. Phys. Rev. E.

[47] Benetti F.P.C., Teles T.N., Pakter R., Levin Y. (2012). Ergodicity Breaking and Parametric Resonances in Systems with Long-Range Interactions. Phys. Rev. Lett..

[48] Campa A., Chavanis P.-H. (2013). Caloric curves fitted by polytropic distributions in the HMF model. Eur. Phys. J. B.

[49] Ewart R.J., Nastac M.L., Schekochihin A.A. (2023). Non-thermal particle acceleration and power-law tails via relaxation to universal Lynden-Bell equilibria. arXiv.

[50] Benetti F.P.C., Ribeiro-Teixeira A.C., Pakter R., Levin Y. (2014). Nonequilibrium Stationary States of 3D Self-Gravitating Systems. Phys. Rev. Lett..

